# Supplementary material for: Development of Methods Derived from Iodine-Induced Specific Cleavage for Identification and Quantitation of DNA Phosphorothioate Modifications
Source: Biomolecules. 2020 Oct 28;10(11):1491. doi: 10.3390/biom10111491 (PMC7692671; doi:10.3390/biom10111491)
Supplement: Supplementary file 1 [file biomolecules-10-01491-s001.zip › Supplementary figure.pdf]

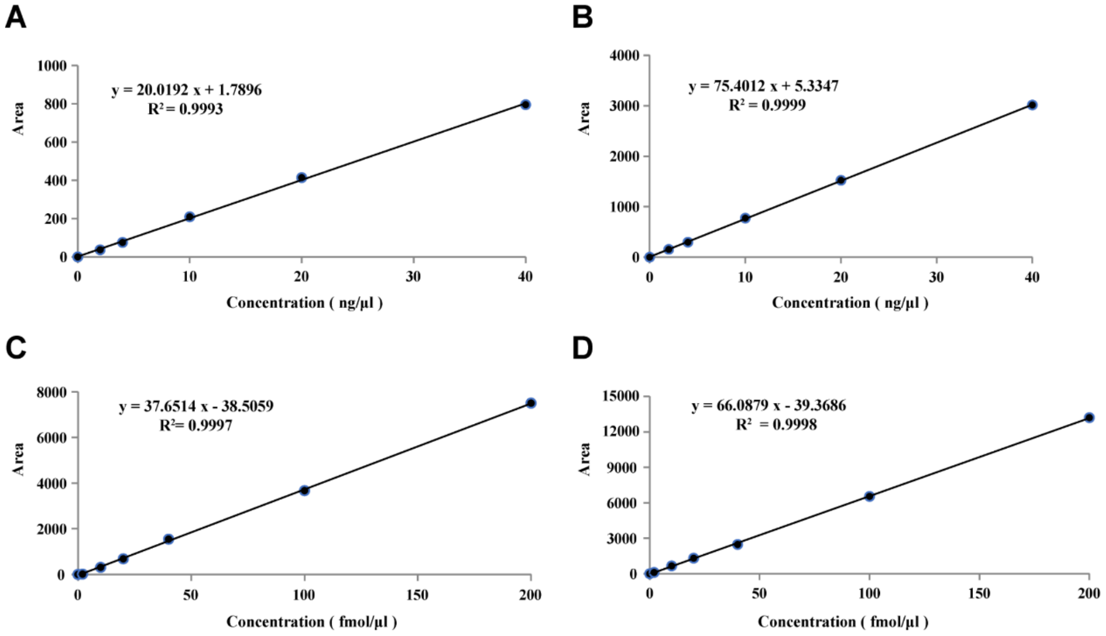

Figure 1. The establishment of standard curve. The standard curve of (A) T and (B) C, and standard curve of (C) GpsA and (D) GpsT.
